# Biochemical, Stabilization and Crystallization Studies on a Molecular Chaperone (PaoD) Involved in the Maturation of Molybdoenzymes

**DOI:** 10.1371/journal.pone.0087295

**Published:** 2014-01-31

**Authors:** Ana Rita Otrelo-Cardoso, Viola Schwuchow, David Rodrigues, Eurico J. Cabrita, Silke Leimkühler, Maria João Romão, Teresa Santos-Silva

**Affiliations:** 1 REQUIMTE, Departamento de Química, Faculdade de Ciências e Tecnologia, Universidade Nova de Lisboa, Caparica, Portugal; 2 Universität Potsdam, Institut für Biochemie and Biologie, Molekulare Enzymologie, Golm, Germany; National Institute for Medical Research, Medical Research Council, London, United Kingdom

## Abstract

Molybdenum and tungsten enzymes require specific chaperones for folding and cofactor insertion. PaoD is the chaperone of the periplasmic aldehyde oxidoreductase PaoABC. It is the last gene in the *paoABCD* operon in *Escherichia coli* and its presence is crucial for obtaining mature enzyme. PaoD is an unstable, 35 kDa, protein. Our biochemical studies showed that it is a dimer in solution with a tendency to form large aggregates, especially after freezing/thawing cycles. In order to improve stability, PaoD was thawed in the presence of two ionic liquids [C_4_mim]Cl and [C_2_OHmim]PF_6_ and no protein precipitation was observed. This allowed protein concentration and crystallization using polyethylene glycol or ammonium sulfate as precipitating agents. Saturation transfer difference – nuclear magnetic resonance (STD-NMR) experiments have also been performed in order to investigate the effect of the ionic liquids in the stabilization process, showing a clear interaction between the acidic ring protons of the cation and, most likely, negatively charged residues at the protein surface. DLS assays also show a reduction of the overall size of the protein aggregates in presence of ionic liquids. Furthermore, cofactor binding studies on PaoD showed that the protein is able to discriminate between molybdenum and tungsten bound to the molybdenum cofactor, since only a Mo-MPT form of the cofactor remained bound to PaoD.

## Introduction

Molybdenum is a transition metal that is incorporated as a biologically active cofactor (molybdenum cofactor, Moco) in a class of widely distributed proteins collectively known as molybdoenzymes [1]. Moco is associated with different redox enzymes and is found in most organisms from bacteria to humans. The metal in Moco is coordinated to the dithiolene of a pterin derivative called molybdopterin [2], [3]. A wide variety of transformations are catalyzed by Moco-containing enzymes at carbon, sulfur, and nitrogen atoms of substrate molecules, which include the transfer of an oxygen group or two electrons to or from the substrate [4]. The molybdenum enzymes are categorized on the basis of the structures of the Moco centers, dividing them into three families, each with a distinct active site structure and a distinct type of reaction catalyzed: the xanthine oxidase family, the sulfite oxidase family, and the DMSO reductase family [4], [5]. The crystal structures of several molybdoenzymes revealed that Moco is deeply buried inside the proteins, at the end of a funnel-shaped passage giving access to the substrate [6]. This implied the requirement of specific chaperones for each molybdoenzyme, to facilitate the insertion of Moco [7].

So far, the best studied molecular chaperone for Moco-binding and insertion is the XdhC protein for xanthine dehydrogenase (XDH) from *Rhodobacter capsulatus* [8]. Investigation of *R. capsulatus* XdhC showed that it binds the Moco and protects it from oxidation until the terminal molybdenum sulfur ligand characteristic for enzymes of the xanthine oxidase (XO) family is inserted [9]. For this reaction, XdhC interacts with a L-cysteine desulfurase that replaces the Mo equatorial oxygen ligand by a sulfido ligand [10]. The sulfur atom for this reaction originates from L-cysteine. After the sulfuration reaction, it is believed that XdhC with its bound sulfurated Moco interacts with the XdhB subunits of the *R. capsulatus* (αβ)_2_ XDH heterotetramer and inserts the mature Moco into this subunit [11]. Thus, XdhC-like proteins perform a number of functions including stabilization of the newly formed Moco, interaction with an L-cysteine desulfurase to ensure that Moco sulfuration occurs as well as interaction with their specific target proteins for insertion of the sulfurated Moco [7]. Thus, the molecular chaperones of the XdhC family were shown to be essential for the maturation of molybdoenzymes of the XO family but are not part of the active holo-molybdoenzymes themselves. Because Moco is deeply buried in the protein, it is also believed that the XdhC proteins may act as chaperones to facilitate the proper folding of the target proteins after Moco insertion. This model implies that molybdoenzymes requiring the sulfurated form of Moco exist in a competent “open” apo-molybdoenzyme conformation until the insertion of sulfurated Moco occurs. After insertion, the protein adapts the final active “closed” conformation that can no longer accept Moco [7].

In *Escherichia coli*, the homologous system to *R. capsulatus* XDH is the aldehyde oxidoreductase PaoABC which is encoded by the *paoABCD* operon [12]. The 135 kDa PaoABC enzyme is located in the periplasm and comprises a noncovalent (αβγ) heterotrimer with a large (78.1 kDa) molybdenum cofactor (Moco)-containing PaoC subunit, a medium (33.9 kDa) FAD-containing PaoB subunit, and a small (21.0 kDa) 2×[2Fe2S]-containing PaoA subunit. PaoD is not a subunit of the mature enzyme, and the protein belongs to the class of XdhC-like molecular chaperones [7], [13]. Analysis of the form of Moco present in PaoABC revealed the presence of a sulfurated molybdopterin cytosine dinucleotide cofactor (MCD) [12], [14]. The PaoD protein was shown to be essential for the insertion of sulfurated MCD into PaoABC and it is expected to play a role similar to that of XdhC with the only difference being that PaoD facilitates sulfuration and insertion of an MCD cofactor rather than an MPT cofactor. Previous studies also showed that PaoD interacts with CTP:molybdopterin cytidylyltransferase MocA and receives the MCD cofactor from it [15]. It is further expected that PaoD is involved in the addition of the terminal sulfido-ligand at the Mo center and the insertion of the mature MCD cofactor into PaoC [13].

In this work, we have further characterized the PaoD protein, purified and stabilized it using ionic liquids (IL). To probe the interaction of PaoD with IL, we have carried out STD-NMR experiments, that are ligand observe experiments, based on the Nuclear Overhauser Effect [16]. Its ability to detect binding of low molecular weight compounds to large biomolecules is well documented [16]–[18]. Additional studies showed that the protein is a dimer in solution which is able to bind Moco. The homologous expression system for PaoD provided us with enough material to perform broad crystallization screenings and we were able to obtain good diffracting crystals of PaoD using ionic liquids.

## Materials and Methods

### 2.1. Purification of *E. coli* PaoD

PaoD was expressed and purified using the procedure described by Neumann *et al.* [15]. *E. coli* BL21(DE3) cells were transformed with plasmid pMN87. For expression, LB medium was inoculated with 1∶100 overnight culture and incubated at 303 K until an OD at 600 nm of 0.3–0.5 was achieved. The expression was induced with 100 mM Isopropyl β-D-1- thiogalactopyranoside. After 5 h growth, the cells were harvested and the cell pellet was resuspended in 50 mM phosphate buffer and 300 mM NaCl, at pH 8.0 (10 mL of buffer per liter of expression culture). Cell lysis was achieved after two passages through a TS Series Benchtop cell disruptor at 1350 bar in the presence of DNase I (1 µg/ml). The cleared lysate was applied to a Ni-tris(carboxymethyl)ethylenediamine (Ni-TED – from Macherey-Nagel) column with 0.4 mL of resin per liter of culture. The column was washed with imidazole solutions at two different concentrations (10 and 20 mM) and PaoD was eluted with 250 mM imidazole in 50 mM phosphate buffer and 300 mM NaCl, pH 8.0. To perform the first crystallization trials the buffer was exchanged to 50 mM phosphate buffer and 300 mM NaCl, pH 8.0 by size exclusion chromatography using a Sephadex G-25 matrix (GE Healthcare). However, due to the high number of salt crystals obtained when phosphate buffer was used, this solution was abandoned in subsequent experiments and replaced by 50 mM Tris-HCl, 1 mM EDTA and 300 mM NaCl, pH 8.0.

The purity of PaoD was determined by SDS/PAGE using Coomassie Brilliant Blue staining. The concentration of the purified PaoD was determined from the absorbance at 280 nm, using an extinction coefficient of 33920 M^-1^cm^-1^. This extinction coefficient was calculated using the bioinformatic tool ProtParam from the ExPASy portal (http://www.expasy.org).

### 2.2. Dynamic Light Scattering (DLS)

To study the effect of differents ionic liquids (IL), 0.4 M C_4_mimCl and C_2_OHmimPF_6_, and additives (1 mM EDTA, 1 mM DTT, 1% Triton X-100 and 300 mM NaCl) in the protein stability, the protein buffer was changed to 50 mM phosphate buffer pH 8.0 or 50 mM Tris-HCl pH 8.0 by size exclusion chromatography using a Sephadex G-25 medium. PaoD at 0.7 mg/ml was incubated with the differents additives for 16 hours at 277 K and, in the case of ionic liquids, for 64 hours at 277 K. Before DLS measurements, all the solutions were centrifuged at 10000 rpm for 30 minutes and filtered throught a microfilter with a pore size of 0.2 µm (Vivaspin 500, Sartorius Stedim Biotech).

The measurements were performed in a SZ-100 Nanopartica Series Instruments (Horiba Scientific, Kyoto, Japan) at 298 K in plastic disposable cells (four opening) and the detector at 90°. The Z_average_ and autocorrelation graph presented were calculated from the average of 4 runs with 120 s each by SZ-100 software for windows.

### 2.3. Moco Binding to PaoD

To study the binding of Mo-MPT, MPT and W-MPT to PaoD free cofactor was prepared using the published procedure in Neumann *et al.* [9]. The cofactor was extracted from purified human sulfite oxidase [19], which was grown with the supplementation of either molybdate or tungstate or in a Δ*moaA E. coli* strain [20]. Freshly purified PaoD (in 100 mM Tris-HCl, 300 mM NaCl, pH 7.2) at 12 µM was incubated in the dark with free Mo-MPT, MPT and W-MPT at different concentrations (from 0 to 48 µM) for 15 min at 277 K. The incubation mixtures were transferred to a Microcon centrifugal filter concentrators (MWCO 10 kDa, Millipore) and centrifuged at 6,000×g for 10 min. To quantify the cofactor content in the assay mixtures, the protein fractions were incubated overnight at room temperature in presence of acidic iodine to convert Mo-MPT, MPT and W-MPT into the oxidized fluorescent product Form A, which can be quantified as described previously [9], [12]. The cofactor in the unbound fraction was determined and related to the total amount of cofactor in the assay mixture to calculate the amount of cofactor bound to the protein. As negative controls, free Mo-MPT, MPT or W-MPT were used in the absence of PaoD and in the presence of BSA, which does not bind any form of the cofactor.

### 2.4. Size Exclusion Chromatography

To analyze the oligomerization state of PaoD, the freshly purified protein was loaded on a Superdex 75 HR10/30 gel filtration column (Amersham Biosciences, Freiburg, Germany) equilibrated in 50 mM Tris-HCl, 300 mM NaCl, pH 8.0. Separation was performed at 283 K and a flow rate of 0.5 mL/min. A BIORAD standard containing gamma-globulin (158 kDa), ovalbumin (44 kDa), myoglobin (17 kDa) and Vitamin B (1.3 kDa) was used to calibrate the column.

### 2.5. STD-NMR Experiments

The STD-NMR experiments were performed with PaoD in Tris-HCl buffer (50 mM Tris-HCl, 1 mM EDTA and 300 mM NaCl, pH 8.0) and two different ionic liquids, [C_4_mim]Cl and [C_2_OHmim]PF_6_. The final concentrations of protein and ionic liquids were *ca* 30 µM and 3 mM, respectively.

All STD-NMR experiments were performed at 310 K in a Bruker Avance III spectrometer operating at 600 MHz, with a 5 mm triple resonance cryogenic probe head. The STD-NMR spectra were acquired with 1024 transients in a matrix with 32 k data points in t2 in a spectral window of 12019.23 Hz centered at 2814.60 Hz. Excitation sculpting with gradients was employed to suppress the water proton signals. A spin lock filter (*T1ρ*) with a 2 kHz field and a length of 20 ms was applied to suppress protein background. Selective saturation of protein resonances (on resonance spectrum) was performed by irradiating at *−*300 Hz using a series of 40 Eburp2.1000 shaped 90° pulses (50 ms, 1 ms delay between pulses), for a total saturation time of 2.0 s. For the reference spectrum (off resonance) the samples were irradiated at 20000 Hz. Proper control experiments were performed with the reference samples in order to optimize the frequency for protein saturation (*−*0.5 ppm) and off resonance irradiation, to assure that the ligand signals were not affected.

The STD effect was calculated by (*I_0_−I_STD_*)/*I_0_*, in which (*I_0_−I_STD_*) is the peak intensity in the STD spectrum and *I_0_* is the peak intensity in the off resonance spectrum. The STD intensity of the largest STD effect was set to 100% as a reference and the relative intensities were determined [16], [18], [21].

### 2.6. Crystallization

Crystallization trials of PaoD were prepared in 50 mM Tris-HCl, 1 mM EDTA and 300 mM NaCl, pH 8.0, and 50 mM phosphate buffer and 300 mM NaCl, pH 8.0, and the protein samples were thawed in the presence of 0.2 or 0.4 M of IL [C_4_mim]Cl as well as of IL [C_2_OHmim]PF_6_. When using either IL at 0.4 M, no precipitation was observed and the protein could be concentrated by centrifugation using a Vivaspin 2 ultrafiltration device (Sartorius Stedim Biotech S.A.).

Protein solutions of 5 mg/mL could thus be obtained when using both IL at 0.4 M and were used for crystallizations assays employing the vapor diffusion method.

Several commercial screenings were tested, namely JBScreen Classic 1–10 (Jena Bioscience), MemStart (Molecular Dimensions) and a 80 conditions in house screen (based on the screen of Jancarik *et al.* [22]). Crystallization drops of 0.2+0.2 µL were set-up using the automatic protein crystallization system Oryx8 (Douglas Instrument). From the several crystallization conditions tested only two gave protein diffracting crystals when the Tris-HCl buffer pH 8.0 was used: in condition 1 protein thawing was done in the presence of 0.4 M [C_4_mim]Cl and ammonium sulphate at 2.2 M was used as precipitating agent; condition 2 contained 12% PEG 4K, 0.1 M Tris-HCl pH 8.5 and 10 mM cysteine and the protein sample had been thawed in the presence of [C_2_OHmim]PF_6_. The reducing agent L-cysteine has proven to be a good choice to improve condition 2 since, in its absence, poorly diffracting crystals were obtained. Other additives from Additive Screen 1 and 2 (Hampton Research) were tried, without success.

**Figure 1:**
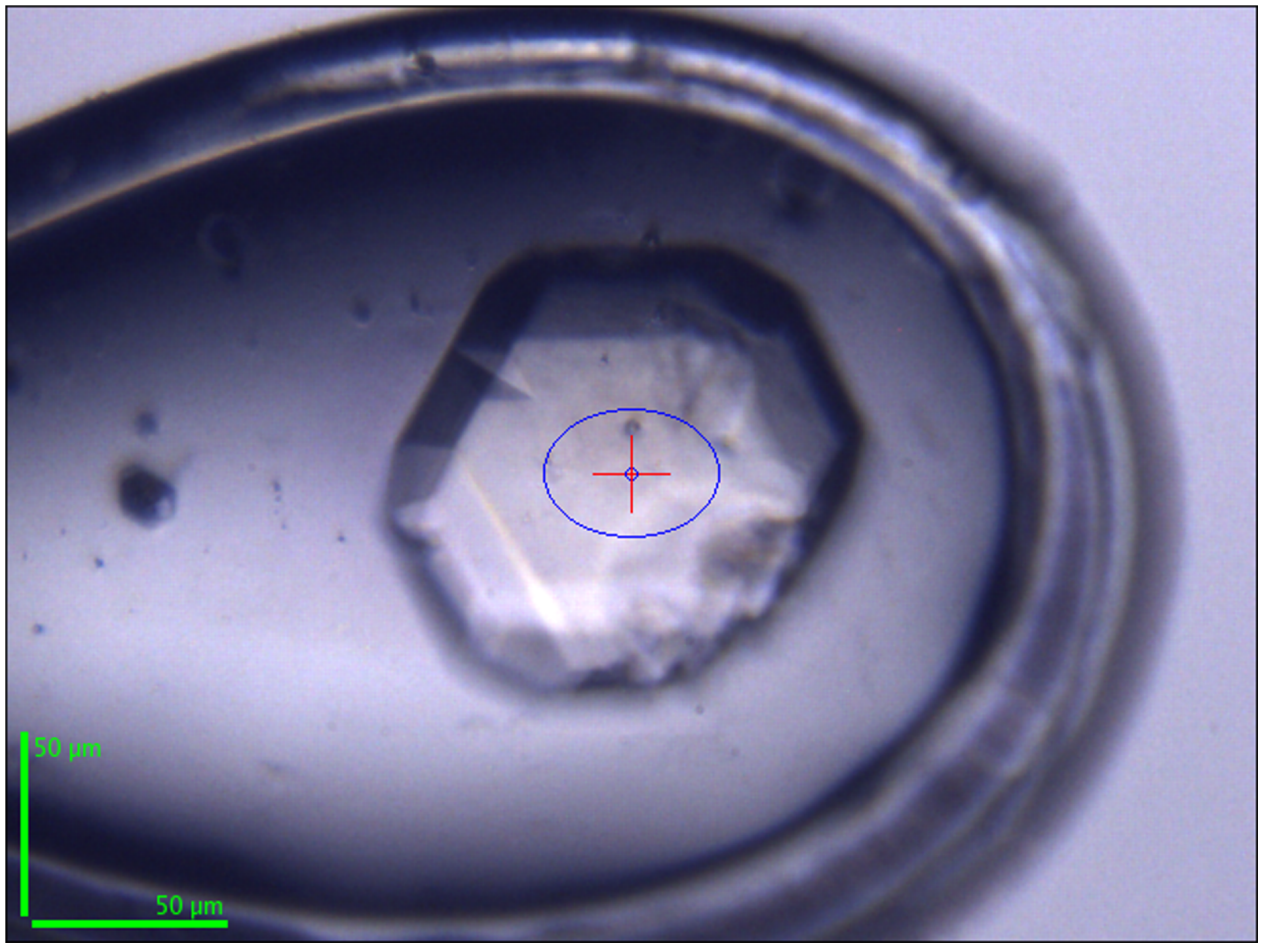
PaoD crystal (0.1×0.08×0.08 mm^3^) obtained with condition 2 (P3_1_21). The protein was thawed in presence of 0.4 M of [C_2_OHmim]PF_6_. The crystallization condition was 12% PEG 4 K, 0.1 M Tris-HCl pH 8.5 and 10 mM cysteine. The same crystal morphology was obtained for the P6_1_22 crystals grown from ammonium sulphate. The image was captured on the synchrotron beamline ID23-1 (ESRF, France).

Both crystallization conditions gave small crystals (0.1×0.1×0.02 mm) with the same morphology within one or two months, respectively, (Figure 1) but were very difficult to reproduce. Scale-up attempts were performed in 24 well crystallization plates (Molecular Dimensions) using 1+1, 1+2 or 2+1 µL drops of protein+precipitant, but with no success.

Nevertheless, synchrotron data collection could be achieved using parathone and glycerol as cryoprotectants. Analysis of the diffraction patterns suggested that parathone is not anadequate cryoprotectant and very poor diffraction was observed. Comparatively, crystals flash frozen after a quick soak with a crystallization solution supplemented with 30% glycerol showed a better diffraction pattern and a complete data set could be collected. Due to the scarcity of available crystals, no other cryo solutions or measurements at room temperature were tried.

**Figure 2:**
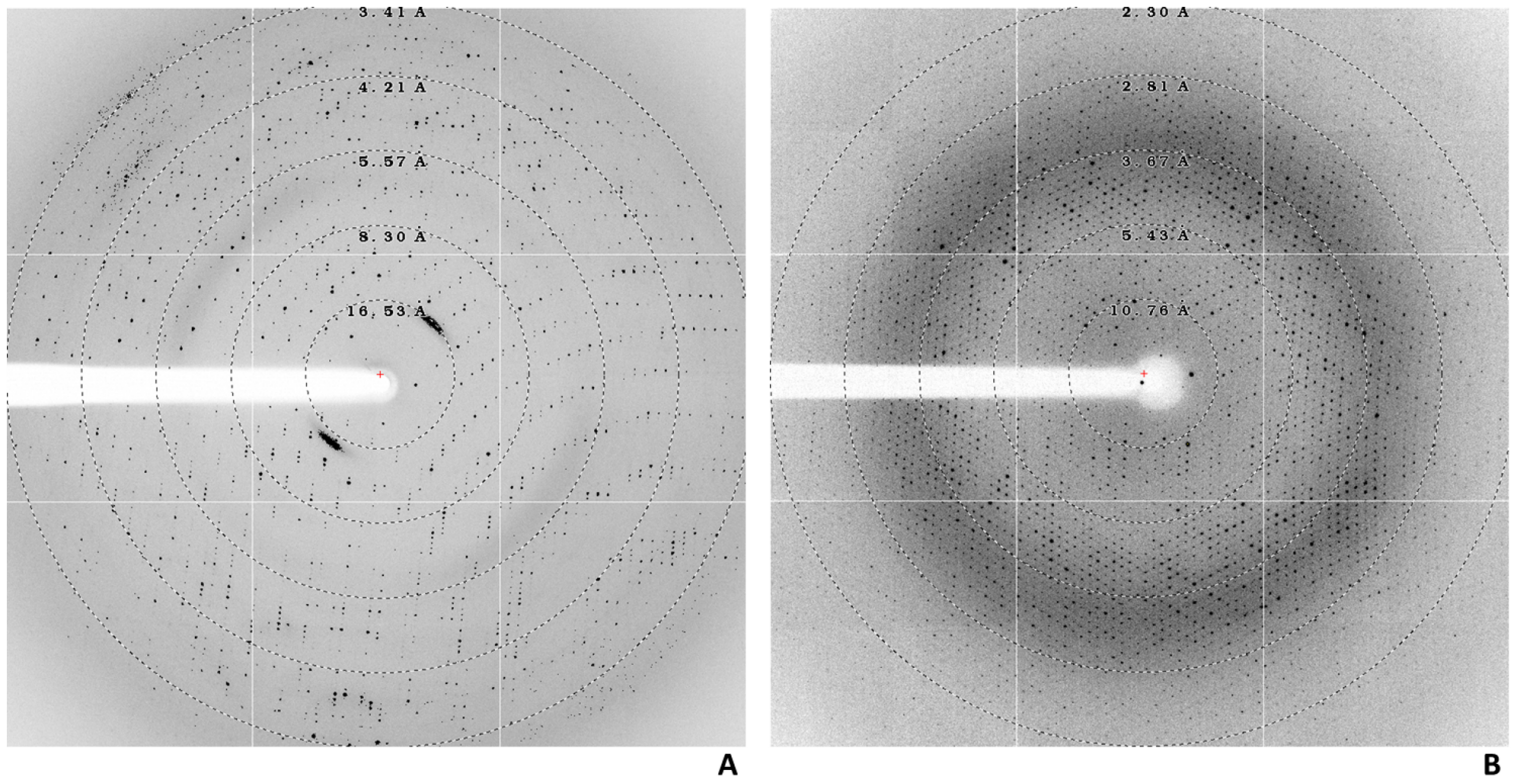
Diffraction pattern of two different PaoD crystal forms. A. Crystal obtained with condition 1 where the protein was thawed in the presence of 0.4[C_4_mim]Cl and ammonium sulphate was used as crystallization agent. B. Crystal obtained with condition 2 where the protein was thawed in the presence of 0.4 M [C_2_OHmim]PF_6_ and using PEG 4 K as crystallization agent. The resolution rings are represented in both images by dashed lines.

The crystals obtained with condition 1, where ammonium sulfate was used as precipitant and the protein was thawed in the presence of 0.4 M of [C_4_mim]Cl, diffracted to a maximum resolution of 3.39 Å (Figure 2A). For the crystallization condition 2 using PEG as the precipitant agent and PaoD thawed in the presence of 0.4 M [C_2_OHmim]PF_6_, crystals diffracted to a maximum resolution of 2.29 Å (Figure 2B).

These data sets were analysed and processed with the programs Mosflm [23], Pointless [24] and Scala [25] that showed that crystals obtained with condition 1 belong to P6_1_22 space group while the ones from condition 2 belong to P3_1_21 space group.

## Results and Discussion

### 3.1. Purification of PaoD and Size Exclusion Chromatography

**Figure 3:**
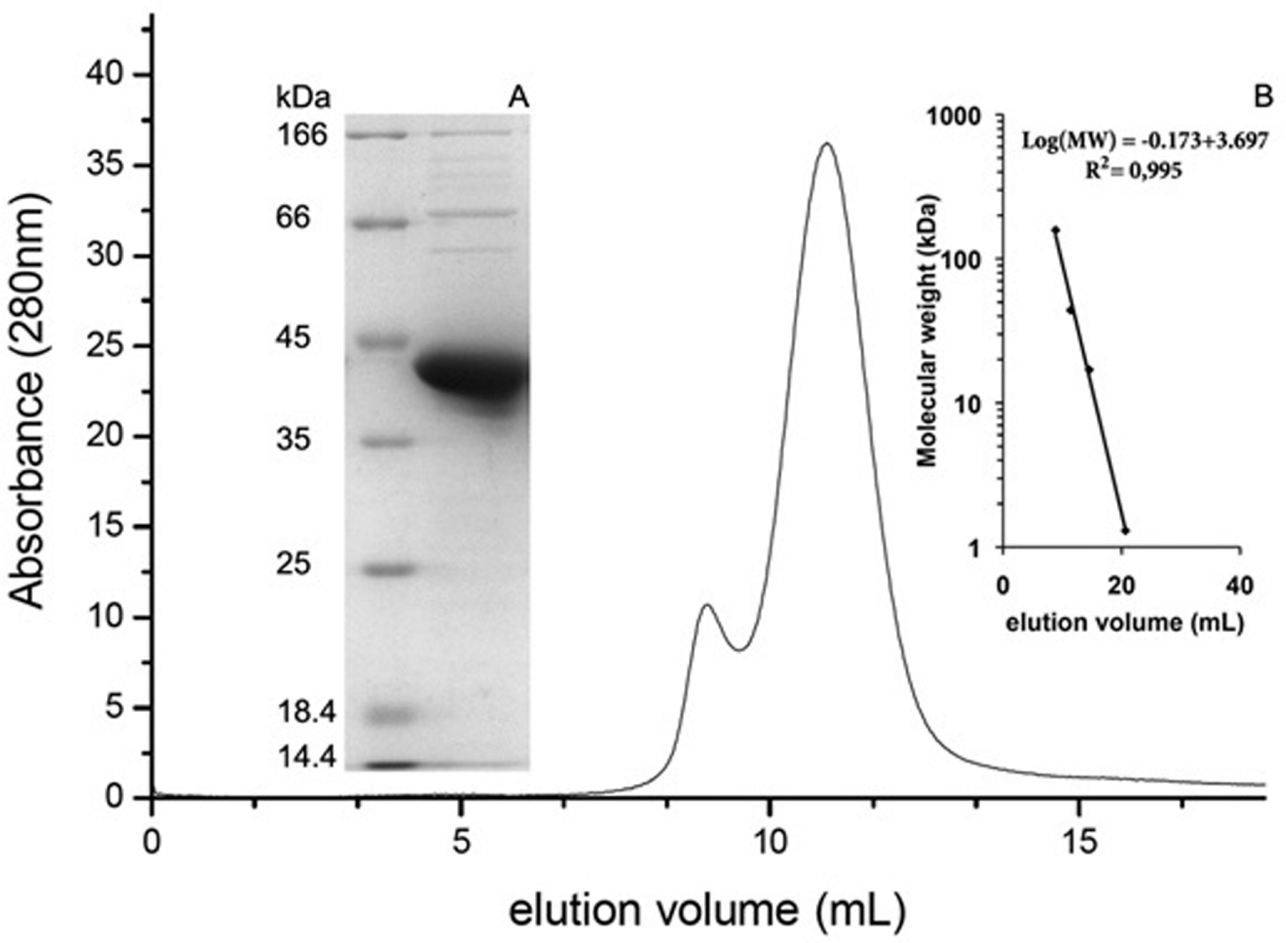
Purification and size exclusion chromatography of PaoD. (A) 12% SDS/PAGE of the purified protein after Ni-TED chromatography. (B) Size exclusion chromatography of PaoD. Freshly purified PaoD was analyzed by analytical size exclusion chromatography in 50 mM Tris-HCl, 300 mM NaCl, pH 8.0 using a Superdex 75 column. Inset: plot of the standard proteins. Size exclusion chromatography markers (Bio-Rad): gamma-globulin (158 kDa), ovalbumin (44 kDa), myoglobin (17 kDa), and vitamin B12 (1.3 kDa).

PaoD was expressed in a homologous expression system in *E. coli* and purified by Ni-TED chromatography. The purified protein showed a single band on Coomassie brilliant blue stained SDS polyacrylamide gel with a molecular mass of 36 kDa, which is in correspondence to the calculated mass of 34.8 kDa (Figure 3B). The protein eluted with a calculated size of 71 kDa from a Superdex 75 size exclusion chromatography column (Figure 3A). The observed elution position of native PaoD shows that it exists in its native state as a dimer in solution. Only a small portion of the protein eluted as a tetramer from the size exclusion column, showing a small tendency to form larger aggregates.

### 3.2. Dynamic Light Scattering (DLS)

The tendency of protein samples to aggregate is a major drawback for crystallization since heterogeneity may hamper good crystal packing and therefore the formation of well ordered crystals. DLS measurements were performed in order to study the effect of different additives (EDTA, DTT, Triton X-100 and NaCl) and ionic liquids ([C_4_mim]Cl and [C_2_OHmim]PF_6_) upon protein stability. Table 1 summarizes the Z_average_ (obtained from the autocorrelation functions present in Figure S1 and Figure S2) and the polydispersity index (PI) obtained for each additive and buffer. The Z_average_ obtained for PaoD in the two buffers tested, 50 mM Tris-HCl (pH 8.0) and 50 mM phosphate (pH 8.0) are high (over 100 nm) indicating that medium-large size aggregates are formed under these conditions. Common additives such as EDTA, DTT or NaCl exhibit either no effect (as for DTT) or drastically decrease protein stability, which is denoted by the very large Z_average_ values observed (500 to 5000 nm). In the presence of Triton X-100 smaller aggregates of PaoD are formed in Tris-HCl buffer, but not in phosphate buffer. The data show that the best additives tested are the two IL since both gave smaller Z_average_ values in the two buffers. The best results are obtained for [C_2_OHmim]PF_6_ in Tris-HCl buffer where Z_average_ values are of 48 nm, even though this corresponds to small size aggregates for a 35 kDa protein as PaoD. The effect of the two IL has been tested with time and, in Tris-HCL buffer, even after 64 hours of incubation, the Z_average_ values observed are within the same order of magnitude. For all additives tested for PaoD in phosphate buffer (50 mM, pH 8.0) the Z_average_ increases in comparison with the results for 50 mM Tris-HCl, pH 8.0 indicating that the latter is better suited for this particular protein. But even in the case of phosphate buffer, the addition of the ILs leads to a reduction of the particles size of *ca* five times when compared with the protein in buffer alone, showing the stabilization properties of the two ILs.

**Table 1:** Comparison between Z_average_ and polydispersity index for PaoD with differents additives and for the two IL, after 16 and 64* hours of incubation.

Buffer	Tris-HCl (50 mM, pH 8.0)	Phosphate buffer (50 mM, pH 8.0)
**Additives**		
0.4 M [C_4_mim]Cl§	105.7±54.8 nm (0.43±0.10)	165.1±38.6 nm (0.38±0.06)
	103.3±13.4 nm (0.50±0.07)*	300.3±46.6 nm (0.43±0.04)*
0.4 M [C_2_OHmim]PF_6_ §	48.8±1.1 nm (0.35±0.03)	116.3±25.3 nm (0.37±0.03)
	89.0±5.5 nm (0.18±0.03)*	576.6±4.5 nm (0.57±0.11)*
1 mM DTT	99.8±10.5 nm (0.58±0.14)	248.7±52.8 nm (0.34±0.02)
1 mM EDTA	5391.3±1625 nm (1.11±0.30)	4234.9±432.8 nm (1.76±0.40)
1% Triton X-100	62.3±4.9 nm (0.56±0.05)	615.5±136.2 nm (0.54±0.07)
300 mM NaCl	2844.2±470 nm (1.81±0.2)	557.0±3.0 mn (0.50±0.02)
300 mM NaCl and 1 mM EDTA	175.8±39.1 nm (0.40±0.05)	–
–	101.7±8.2 nm (0.47±0.05)	630.4±60.5 nm (0.53±0.19)

(Data in parenthesis correspond to the polydispersity index).

§ Assays performed with buffer supplemented with 300 mM NaCl. The Tris-HCl pH 8.0 buffer was supplemented with 1 mM of EDTA.

In the absence of ionic liquids the formation of large aggregates is obvious since protein precipitation is clearly visualized after 24 hours at 277 K. The fact that the presence of these stabilizing molecules seems to reduce the amount of protein aggregates in solution could be the reason for the success in crystallization under these conditions.

### 3.3. Cofactor Binding of PaoD

It has been reported previously that PaoD is essential for MCD sulfuration and MCD insertion into PaoABC [13]. Here, we were interested to analyze the Moco-binding properties of PaoD further. Since the MCD-form of Moco is not stable in its isolated state, we analyzed the binding of Mo-MPT, W-MPT and MPT to PaoD. Mo-MPT, W-MPT and MPT were extracted from purified human sulfite oxidase, grown under different conditions and incubated with PaoD. Excess of cofactor was removed by ultrafiltration and the cofactor of the protein-bound fraction was converted into the oxidized fluorescent product Form A that was further quantified by HPLC. The obtained chromatograms showed that Mo-MPT and MPT was bound to PaoD. In the PaoD protein samples incubated with W-MPT and in the BSA samples, no peaks were observed. The obtained results show that binding of Mo-MPT and MPT occurs with PaoD but not with W-MPT, suggesting that the protein is able to discriminate between the different metals bound to the MPT-core. Determination of the exact stoichiometry of the cofactor binding to PaoD was not possible due to the intrinsic instability of the protein since, even at low protein concentration (12 µM), precipitation occurred overnight. Additionally, PaoD binds the MCD form of the cofactor after its formation by the MocA protein. Unfortunately, it is not possible to extract MCD in a stable form from proteins, thus, MCD-binding to PaoD was not monitored.

### 3.4. Interaction of Ionic Liquids with PaoD and STD-NMR Data

Stability is a detrimental characteristic for any structural study of biomacromolecules. PaoD is an unstable, medium size protein that tends to aggregate, especially after a cycle of freezing/thawing. As mentioned previously, dynamic light scattering (DLS) assays showed the formation of medium and large aggregates, even when using freshly purified protein (see the results on section 3.2). The low expression levels together with the difficulty in achieving high protein concentrations compromises crystallization experiments. Attempting to overcome this problem, several compounds commonly known as protein stabilizers, have been used, such as DTT, Triton X-100 and EDTA. The best results were obtained when the protein was in the presence of 300 mM NaCl and 1 mM EDTA in Tris-HCl pH 8.0 with ionic liquids (see section 3.2).

In recent years, there has been an increasing attention in the use of ionic liquids to increase the stability of proteins [26]–[28] as well as crystallization additives [29], [30]. Therefore, we have tested ILs such as [C_4_mim]Cl, [C_2_OHmim]PF_6_ and [C_4_mim]MEES in the attempt to obtain more concentrated and stable PaoD solutions. Since protein precipitation occurs upon thawing of the sample, we decided to add the IL during this process, to a final concentration of either 0.2 or 0.4 M. In this way, the protein would thaw in the presence of the IL, which would help preventing its precipitation and the procedure turned out to be very successful for [C_4_mim]Cl and [C_2_OHmim]PF_6_ at 0.4 M. In order to understand the putative interactions between the protein and the IL, responsible for the observed increase in stabilization, STD-NMR experiments were performed.

**Figure 4:**
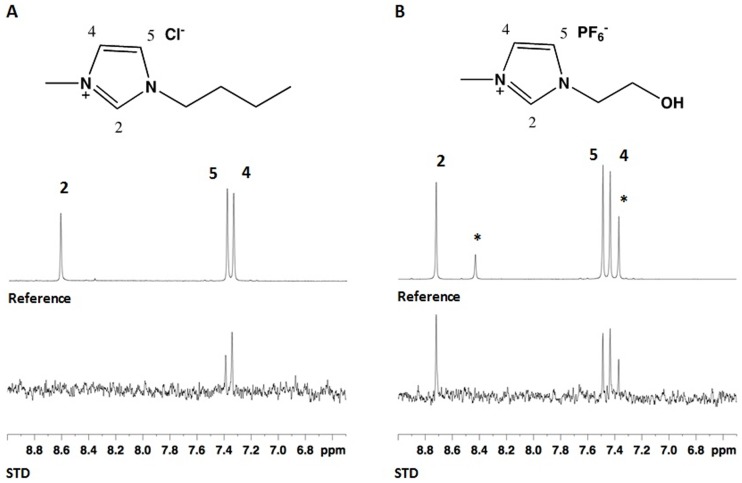
Expansion of the aromatic region of (A) the reference and the STD NMR spectrum obtained with [C_4_mim]Cl and (B) the reference and the STD NMR spectrum obtained with [C_2_OHmim]PF_6_. *Marks the peaks of imidazole present in the protein sample.

As described in the literature for other proteins [30], either the anionic or cationic counterpart of the IL can stabilize proteins through protein-ion interactions. STD-NMR is a robust method that can provide information about intermolecular interactions from the viewpoint of the small molecule, allowing the characterization of low-affinity interactions between small molecules and (bio)macromolecules [18], [31]. The STD-NMR experiment is based on a transfer of saturation from the protein to the ligand, which is in a much higher concentration compared to the protein. By measuring this saturation transfer one can identify the existence of a protein-ligand interaction and determine which part of the ligand is responsible for this interaction. STD-NMR experiments were performed with [C_2_OHmim]PF_6_ and [C_4_mim]Cl, as described in the experimental section, and the resulting STD-NMR spectra are presented in Figure 4.

As can be seen in Figure 4, STD responses from the aromatic protons of the cation were detected both for [C_2_OHmim]PF_6_ and [C_4_mim]Cl. This result is a clear indication that there is an interaction between the protein and the ILs in solution. Since the pI of the protein, determined by sequence analysis using the ExPASy portal (http://www.expasy.org), is around 6.5 and at the working pH (8.5) the protein is negatively charged, we anticipate that electrostatic forces can be among the main driving force for the interaction. More interesting is the fact that no STD signals from the methyl group or alkyl protons of the imidazolium ring substituents could be detected. The absence of signals from these moieties and the comparison of the intensities between the reference and the STD spectra seem to suggest that the interaction between the IL cation and the protein has some degree of specificity and directionality. This directionality should be similar to the one found between the IL cation and the anion, which occurs preferentially with the more acidic ring protons 2, 4 and 5, that are able to participate in hydrogen bonds.

The major difference between the two ILs is the absence of response from the more acidic proton 2 in the case of [C_4_mim]Cl and an overall higher relative STD intensity for [C_2_OHmim]. At this point we are not able to explain this result completely. However, the charge dispersion and polarity of the cation may be responsible for the differences. For weak binding ligands the intensity of the STD can provide qualitative information concerning the relative affinity and specificity of the different ionic liquids if the experiments are performed under the exact same conditions of concentration and IL:protein ratio. Under these conditions a higher intensity of the STD response can be due to a higher affinity, a less specific interaction or both since these factors will increase the efficiency of the saturation transfer process. Since [C_2_OHmim] is smaller and more polar than [C_4_mim] this can favour a stronger and less specific interaction with the protein that would explain the higher intensity of the STD response for [C_2_OHmim] when compared to [C_4_mim]. Therefore [C_2_OHmim] can present a more extended interaction surface than [C_4_mim], which, most likely, is contributing for an increase in PaoD stability, thus explaining why protein precipitation is avoided upon thawing in the presence of this IL. The anion may also play a role in the interaction, since for the two ILs different degrees of solvation of the ion pairs are expected in the buffer system. This can have consequences in the availability of the cation to participate in interactions with the protein but a more extended investigation with different combinations of cations/anions is required to clarify the relative contribution of the anion [32].

Since the STD-NMR experiment is a ligand observe experiment, the results obtained do not allow to identify the moieties of the protein responsible for the interaction. However, the pattern of the interaction observed suggests that charged negative side chain residues, prone to participate in hydrogen bonds, could be responsible for the directionality of the interaction observed, explaining the lack of STD response from the other IL moieties.

### 3.5. Crystallographic Data

The crystallization trials of PaoD were performed with a protein solution concentrated to approximately 5 mg/mL (in storage buffer 50 mM Tris-HCl, 300 mM NaCl, 1 mM EDTA or 50 mM phosphate buffer and 300 mM NaCl, pH 8.0) in the presence of ionic liquids. However, due to the high number of salt crystals obtained when phosphate buffer was used, this solution was abandoned in subsequent experiments. After performing multiple screenings, it was possible to obtain single, well diffracting crystals from two different crystal forms using two different crystallization agents: condition 1 with 2.2 M of ammonium sulphate and the protein thawed in the presence of [C_4_mim]Cl and condition 2 with 12% PEG 4 K, 0.1 M Tris-HCl pH 8.5 and 10 mM cysteine, and the protein thawed with [C_2_OHmim]PF_6_ (cf. Table 2).

**Table 2:** Data collection statistics for PaoD crystals. (Data in parenthesis correspond to the highest resolution shell.).

Crystal form	1	2
Crystallization condition	2.2 M Ammonium sulfate	12% PEG 4 K, 0.1 M Tris-HCl pH 8.5 and 10 mM cysteine
Ionic liquid in protein solution	0.4 M [C_4_mim]Cl	0.4 M [C_2_OHmim]PF_6_
Wavelength (nm)	0.975	0.979
Space group	P6_1_22	P3_1_21
Unit-cell parameters (Å)	a = b = 144.44, c = 240.48 Å	a = b = 106.41, c = 237.41 Å
Matthews parameter (Å^3^/Dalton)	2.59 (4 molecules/AU)	2.77 (4 molecules/AU)
No. observed reflections	285511 (36291)	356767 (17503)
No. unique reflections	21154 (2917)	70445 (4395)
Resolution limits (Å)	86.59–3.39 (5.57–3.39)	60.04–2.29 (2.35–2.29)
Completeness (%)	99.0 (96.8)	99.8 (97.4)
R_pim_ (%)*	4.0 (15.7)	4.1 (10.7)
R_meas_ (%)^#^	27.1 (94.0)	9.5 (22.5)
<I/σ(I)>	14.5 (5.6)	9.7 (3.2)
Multiplicity	13.5 (12.4)	5.1 (4.0)
Average mosaicity	0.77	0.45

*
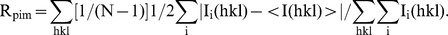

#
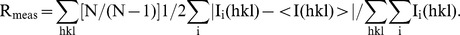

Both crystal forms were measured using synchrotron radiation, at ID23-1 beamline at the ESRF (Grenoble, France). Crystals obtained with condition 1 diffract up to 3.39 Å and belong to space group P6_1_22 with cell constants a = b = 144.44, and c = 240.48 Å. A second crystal form was obtained with condition 2. The crystals diffracted up to 2.3 Å resolution and a higher resolution data set was collected. They belong to the P3_1_21 space group, with cell constants a = b = 106.41, and c = 237.41 Å (data collection statistics in Table 2).

The Matthews coefficient and the solvent content of the crystals were calculated for the two data sets. The obtained values are very similar (2.59 and 2.77 Å^3^/Da for condition 1 and condition 2 crystals, respectively) and suggest the presence of four molecules in the asymmetric unit, with ca 50% of solvent. Taking into account the size exclusion chromatography results that indicate that PaoD is a dimer in solution, the arrangement of the four molecules in the asymmetric unit should correspond to a dimer of functional dimers. Structure determination has not yet been accomplished due to the lack of a proper homology model. Selenomethionine expression is currently under way for Se-SAD experiments and, in parallel, attempts are being made to prepare heavy atoms derivatives.

### Conclusions

The molecular chaperone PaoD is essential for the maturation of the periplasmic aldehyde oxidase PaoABC [12]. PaoD has been purified as a dimeric protein and could be successfully stabilized and concentrated in the presence of specific ionic liquids (IL) ([C_4_mim]Cl and [C_2_OHmim]PF_6_). In order to characterize the interaction of the IL moieties with the protein, STD-NMR studies were carried out. The results suggest some degree of directionality and specificity in the interaction IL-protein. The use of ionic liquids for the stabilization and concentration of the chaperone PaoD proved also to be essential for the crystallization assays. The stabilization effect of ILs reduced the precipitation and the size of aggregates (DLS assays) during the thawing process and allowed the protein to be concentrated making it possible to obtain well diffracting crystals. This constitutes an important step towards the structural elucidation of this molecular chaperone.

Furthermore, binding assays revealed some degree of specificity of PaoD regarding metal incorporation. When exposed to W-MPT, Mo-MPT and MPT, the protein does not bind the W-containing cofactor. This result suggests that PaoD can distinguish between the two metals and selectively incorporate molybdenum. Since PaoD is the chaperone responsible for the sulfuration and insertion of the MCD cofactor into the apo-form of PaoABC, molybdenum has to be bound to the MPT-CMP core prior to binding to PaoD.

## Supporting Information

Figure S1 Autocorrelation graph for PaoD in presence of different ionic liquids after 16 hours of incubation. (A) Protein in 50 mM Tris-HCl, 300 mM NaCl and 1 mM EDTA pH 8.0 (blue), with 0.4 M [C_4_mim]Cl (black) and 0.4 M [C_2_OHmim]PF_6_ (orange). (B) Protein in 50 mM Phosphate buffer and 300 mM NaCl pH 8.0 (blue), with 0.4 M [C_4_mim]Cl (black) and 0.4 M [C_2_OHmim]PF_6_ (orange).
(TIF)

Figure S2 Autocorrelation graph for PaoD in presence of different additives after 16 hours of incubation. (A) Protein in 50 mM Tris-HCl pH 8.0 (black), with 1 mM DTT (grew), 300 mM NaCl (pink), 1 mM EDTA (blue) and 1% Triton X-100 (green). (B) Protein in 50 mM Phosphate buffer pH 8.0 (black) with 1 mM DTT (grew), 300 mM NaCl (pink), 1 mM EDTA (blue) and 1% Triton X-100 (green).
(TIF)
