# Supplementary figures and images for: Biochemical, Stabilization and Crystallization Studies on a Molecular Chaperone (PaoD) Involved in the Maturation of Molybdoenzymes
Source: PLoS One. 2014 Jan 31;9(1):e87295. doi: 10.1371/journal.pone.0087295 (PMC3909100; doi:10.1371/journal.pone.0087295)

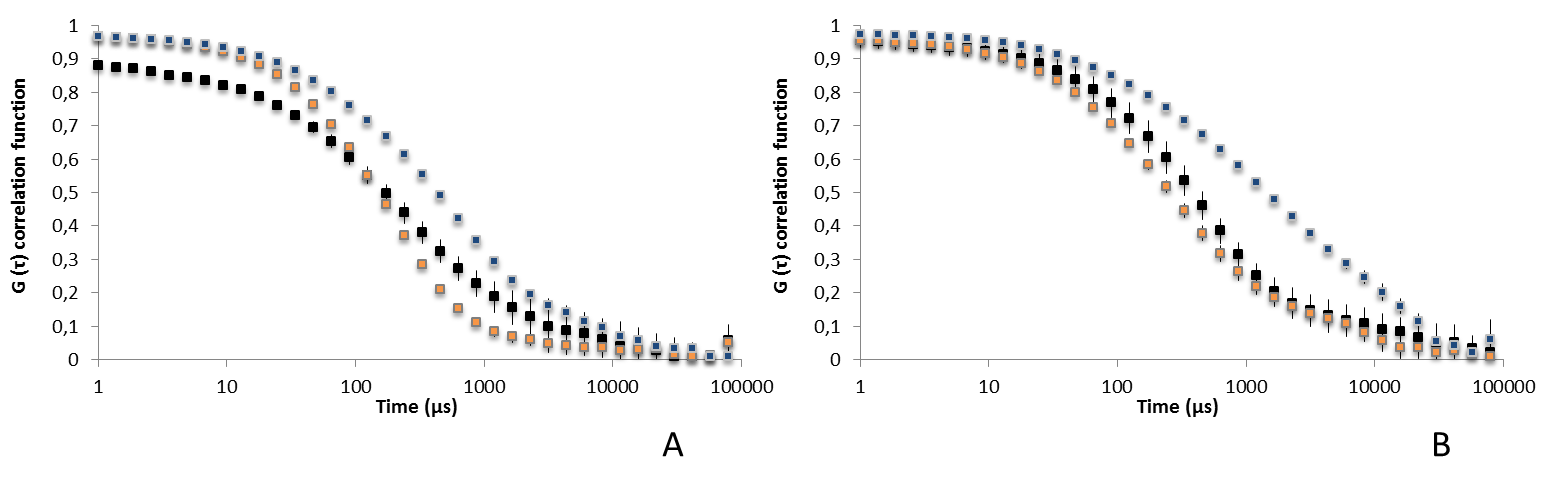

Supplement: Figure S1 — Autocorrelation graph for PaoD in presence of different ionic liquids after 16 hours of incubation. (A) Protein in 50 mM Tris-HCl, 300 mM NaCl and 1 mM EDTA pH 8.0 (blue), with 0.4 M [C4mim]Cl (black) and 0.4 M [C2OHmim]PF6 (orange). (B) Protein in 50 mM Phosphate buffer and 300 mM NaCl pH 8.0 (blue), with 0.4 M [C4mim]Cl (black) and 0.4 M [C2OHmim]PF6 (orange). (TIF) [file pone.0087295.s001.tif]

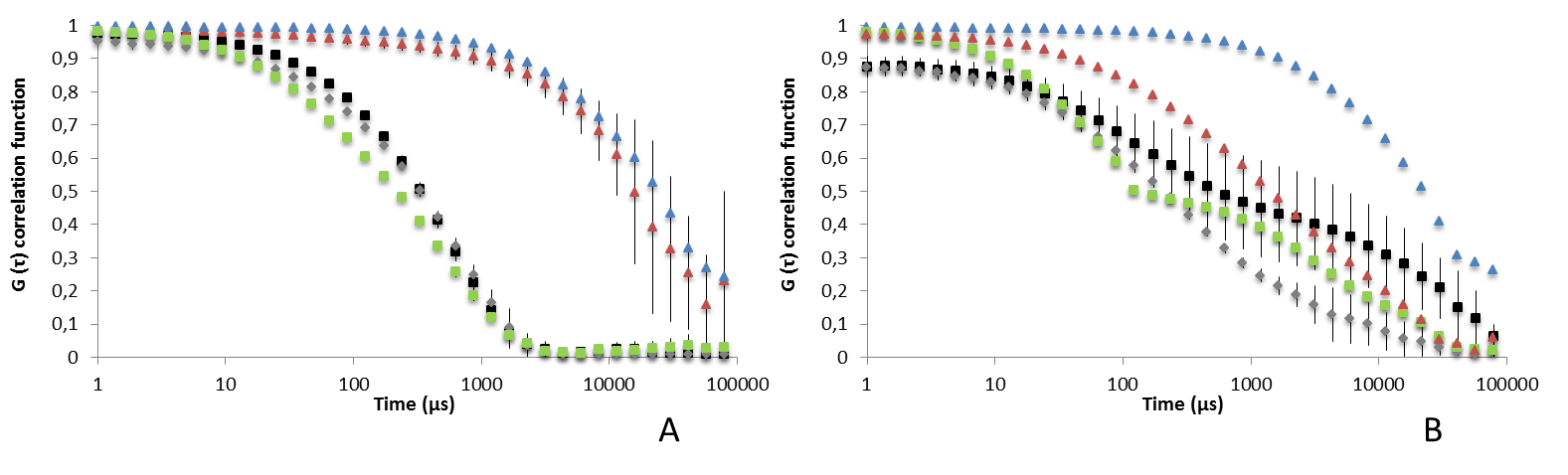

Supplement: Figure S2 — Autocorrelation graph for PaoD in presence of different additives after 16 hours of incubation. (A) Protein in 50 mM Tris-HCl pH 8.0 (black), with 1 mM DTT (grew), 300 mM NaCl (pink), 1 mM EDTA (blue) and 1% Triton X-100 (green). (B) Protein in 50 mM Phosphate buffer pH 8.0 (black) with 1 mM DTT (grew), 300 mM NaCl (pink), 1 mM EDTA (blue) and 1% Triton X-100 (green). (TIF) [file pone.0087295.s002.tif]
